# Quantification and Confirmation of Fifteen Carbamate Pesticide Residues by Multiple Reaction Monitoring and Enhanced Product Ion Scan Modes via LC-MS/MS QTRAP System

**DOI:** 10.3390/molecules23102496

**Published:** 2018-09-29

**Authors:** Ying Zhou, Jian Guan, Weiwei Gao, Shencong Lv, Miaohua Ge

**Affiliations:** Jiaxing Center for Disease Control and Prevention, Zhejiang 314050, China; zhouyingand@sina.cn (Y.Z.); jguan001@sina.com (J.G.); dannyday@126.com (W.G.); thestorm2008@126.com (S.L.)

**Keywords:** carbamates, multiple reaction monitoring (MRM), enhanced product ion (EPI), mass fragmentation, confirmatory method, pesticide residues

## Abstract

In this research, fifteen carbamate pesticide residues were systematically analyzed by ultra-high performance liquid chromatography–quadrupole-linear ion trap mass spectrometry on a QTRAP 5500 system in both multiple reaction monitoring (MRM) and enhanced product ion (EPI) scan modes. The carbamate pesticide residues were extracted from a variety of samples by QuEChERS method and separated by a popular reverse phase column (Waters BEH C18). Except for the current conformation criteria including selected ion pairs, retention time and relative intensities from MRM scan mode, the presence of carbamate pesticide residues in diverse samples, especially some doubtful cases, could also be confirmed by the matching of carbamate pesticide spectra via EPI scan mode. Moreover, the fragmentation routes of fifteen carbamates were firstly explained based on the mass spectra obtained by a QTRAP system; the characteristic fragment ion from a neutral loss of CH_3_NCO (−57 Da) could be observed. The limits of detection and quantification for fifteen carbamates were 0.2–2.0 μg kg^−1^ and 0.5–5.0 μg kg^−1^, respectively. For the intra- (*n =* 3) and inter-day (*n =* 15) precisions, the recoveries of fifteen carbamates from spiked samples ranged from 88.1% to 118.4%, and the coefficients of variation (CVs) were all below 10%. The method was applied to pesticide residues detection in fruit, vegetable and green tea samples taken from local markets, in which carbamates were extensively detected but all below the standard of maximum residue limit.

## 1. Introduction

Carbamate pesticides, namely, N-methyl carbamate esters not only share with organophosphates the capacity to kill insects by inhibiting the enzyme acetylcholinesterase (AChE) but also show lower toxicity to human being [1,2,3]. Gradually, they have become one kind of most popular pesticides in agriculture to guarantee food production. However, the improper use of carbamate pesticides have adversely affected food safety g [4,5]. Carbamate pesticide residues are known to be frequently present in fruits, vegetables and green teas on the market at levels close to or below the standard of maximum residue limit [6]. The legitimate content has brought chronic and continuous intake of carbamate residues that could be relevant to multiple ailments and further pose a threat to public health [7,8,9]. Meeting the challenge of many samples in daily work, developing a best concise, highly specific and sensitive analytical method to detect the carbamate residues in diverse samples from the area of food safety is important.

The challenge to modify this analytical method could be ascribed to two main factors: the existence of a range of carbamate pesticides and samples with complex composition that depend on the instrumental analysis and the sample pretreatment. With the efforts of researchers for half a century, the sample pretreatment of pesticide residues has been developed from Soxhlet extraction, liquid–liquid partition chromatography and column chromatography to solid-phase extraction, solid-phase micro-extraction, molecularly imprinted solid-phase extraction, super critical fluid extraction, matrix solid phase dispersion and QuEChERS (Quick, Easy, Cheap, Effective, Rugged, and Safe) methods [10,11,12]. Developed in 2003 by Anastassiades, QuEChERS sample preparation has been readily accepted by many pesticide residue analysts over the years [13,14]. In the section of instrumental analysis, a literature survey (Table 1) shows several methods for the detection of carbamate pesticide residues have been developed at low concentration levels in various samples, such as electrochemical detection [15], liquid chromatography [16,17,18,19,20,21,22,23,24], high-performance thin-layer chromatography [25], liquid chromatography with post-column fluorescence derivatization [26,27] and liquid chromatography with different mass spectrometry [28,29,30,31,32]. Of all the instrumental methods, the LC-MS system that combines the separation ability of liquid chromatography along with the sensitivity and specificity of detection from mass spectrometry and abandoned the additional derivation procedure of whole analysis process has gone mainstream for pesticide residue analysis. Among the different mass spectrometers, the ion trap triple quadrupole mass spectrometer allows for multiple reaction monitoring (MRM) scan modes is the ascendant instrument to quantify the residues of pesticides at trace amount. In MRM mode, the selected precursor ion of relevant compound is pre-screened in the first quadrupole (Q1), dissociated in the second quadrupole (Q2) and identified and quantified in the third quadrupole (Q3). During the analysis procedure, both Q1 and Q3 are set at several specific masses, allowing only those distinct fragment ions from the certain precursor ions to be detected. The setting of certain precursor ions and specific masses results in increased sensitivity and the structural specificity of the analyte [33,34].Compared with traditional triple quadrupole mass spectrometry, the Triple Quadrupole Linear Ion Traps system (QTRAP, AB SCIEX) equips an extra linear ion trap (LIT) located in the third quadrupole set and represents the new generation of LC-MS/MS system with unique scan mode of multiple reaction monitoring–information dependent acquisition–enhanced product ion (MRM-IDA-EPI) scan mode, which could maximize the information of the analytes in a single run, including selected ion pairs, retention time, relative intensities and the mass spectra. In this mode, the ions of analytes enter Q3 and are stopped from leaving this region by the changing of voltages. Then, the trap is allowed to fill with target ions with a set amount of time. The voltages are changed to stop any more ions from entering. Finally, the packet of ions is scanned out from the trap in a controlled manner. The target analytes are quantified as ever, and further identified on the automatically acquired MS/MS spectra while the precursor ions intensity exceeds the fixed area threshold setting. In particular, the linear ion trap could keep on capturing the interested precursor ions and product ions for a few milliseconds. This accumulation signifies a 500-times increase in the scanning sensitivity and brings a high intensity of the mass spectra recorded in EPI mode. It double checks the doubtful samples by comparing the acquired mass spectra with the mass spectral library built from standards [35]. This study developed a new analytical strategy to combine QuEChERS sample preparation and QTRAP LC-MS/MS system to improve the efficiency of quantification and confirmation of carbamate residues while ensuring high degree of reproducibility. In addition, this method should be stable enough to avoid false negative or false positive results.

## 2. Results and Discussion

### 2.1. Optimization of Sample Preparation

**Figure 1:**
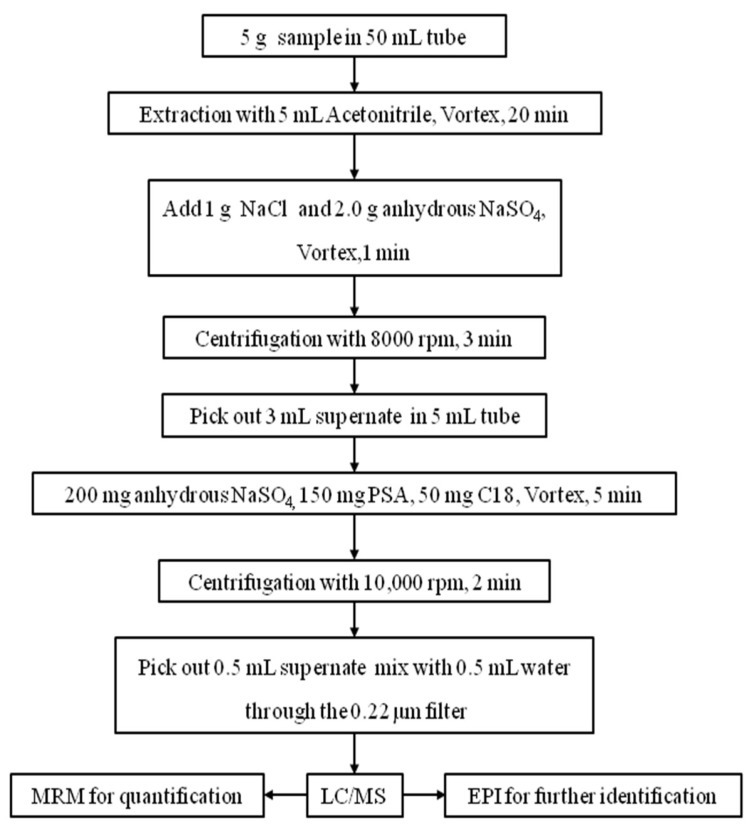
Scheme of analysis for the simultaneous determination and identification of fifteen carbamate pesticide residues in multi-matrices.

As shown in Figure 1, the scheme of analysis method, extraction process and purification process are the two main components of the QuEChERS pretreatment. In this study, acetonitrile with different concentrations of formic acid supplement was compared for extraction efficiency. However, acetonitrile and acidified acetonitrile did not show any obvious distinction in the recovery experiment. Thus, it was more convenient to adopt acetonitrile in the extraction process. In the purification process, 1.0 g of sodium chloride and 2.0 g of anhydrous sodium sulfate were applied to separate the aqueous solution and acetonitrile part. In this study, the traditional anhydrous magnesium sulfate was replaced by anhydrous sodium sulfate because of the heat release in the moisture absorbing process. In the purification process, the commonly used cleaning agents are ethylenediamine-N-propylsilane (PSA), graphitized carbon black (GCB) and C18. PSA is pertinent to organic acids, some pigments, and some sugars and fats; GCB has a strong adsorption effect on pigments; and C18 aims to remove non-polar impurities such as fats. Different proportions of PSA, GCB, and C18 were combined to find the best recipe with the best average recoveries. It was proventhat the combination of 50 mg C18 and 150 mg PSA as cleaning agent was most suitable for determining quantitatively carbamate residues in multi-matrices by recovery tests and samples determination (Figure S1).

### 2.2. Optimization of Mass Spectrometry and Chromatographic Conditions

To confirm the ion pairs for quantification, the mixed standard solution of fifteen carbamate pesticides with a concentration of 100 µg L^−1^ was infused into the QTRAP mass spectrometer at a flow rate of 7 µL min^−1^ to obtain automatic analytes optimization by the ESI positive mode. In the optimal mass spectrometry conditions, all carbamates except tsumacide acquired two pairs of optimum ions for quantification and identification. 

**Figure 2:**
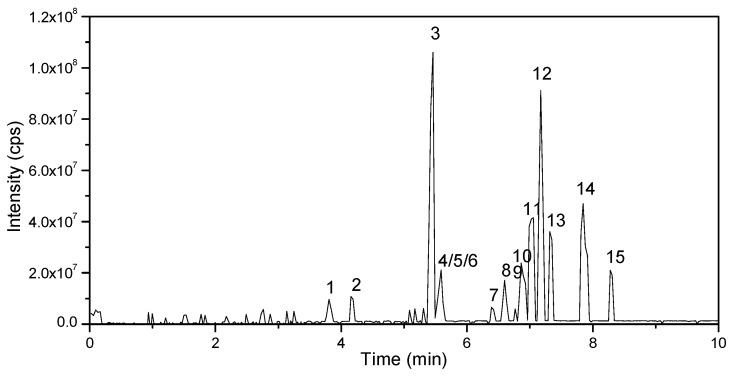
LC-MS/MS chromatograms of fifteen carbamate pesticides (50 ng mL^−1^).

For the high sensitivity, symmetric shape of the ionic peaks and minimal retention time, this study examined the elution type, flow rate, gradient and the type of the chromatographic column. Once the most general chromatographic column C18 was decided, the separation and ionization of the analytes were mainly affected by the compositions of the elution type and the elution gradient. Therefore, several classical compositions of the mobile phase were performed including methanol, acetonitrile, water and water with ammonium acetates or formic acid. Finally, water and acetonitrile, both supplemented with 0.1% formic acid, were chosen as the optimal mobile phases. The final gradient elution with the total flow rate of 0.3 mL/min was as follows: 0–2.5 min, 15% A; 2.5–5 min, 15–50% A; 5–7 min, 50% A; 7–8 min, 50–15% A and 8–10 min, 15% A. The column oven was maintained at 40 °C and the injection volume was 5 µL. The representative LC-QTRAP-MS/MS chromatograms were merged (Figure 2). The retention time (RT) and MS information for each analyte including precursor and product ions, DP and CE are shown in Table 2. 

The EPI survey scans in IDA experiment should be triggered when the ionic intensity exceeded the threshold of 1000 cps. The total scan time (including pauses) was 1.08 s for all MRM transitions. The range of the mass spectra was set between 50 and 300 amu with a scan rate of 10,000 Da s^−1^. The mass spectra of fifteen carbamates and their characteristic fragmentations are shown in the Supplementary Materials.

### 2.3. Fragmentation Manner of Carbamate Pesticides

**Figure 3:**
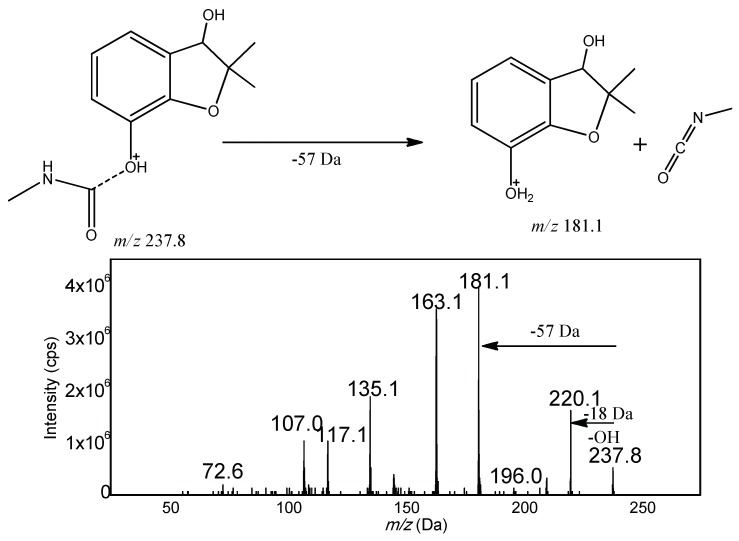
Product ion spectrum and probable fragmentation routes of Carbofuran-3-hydroxy.

**Figure 4:**
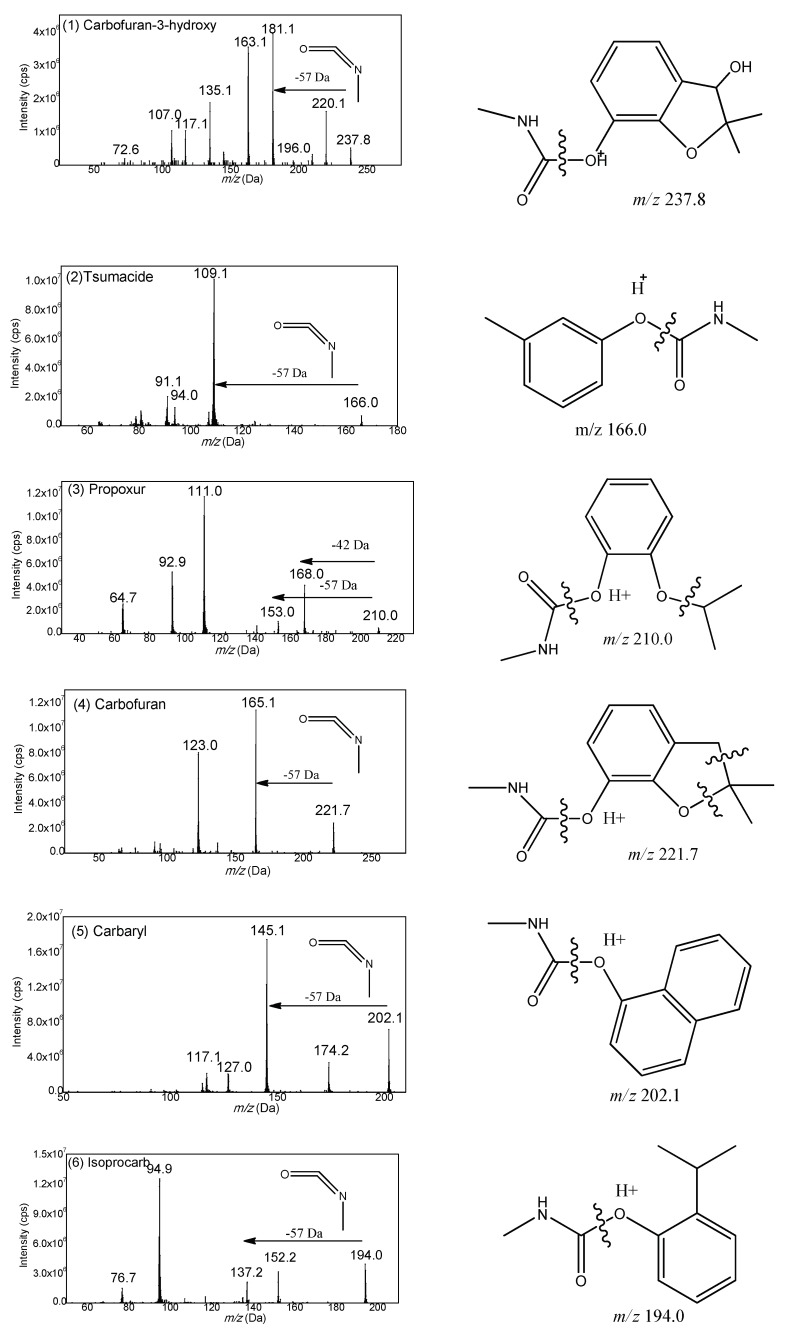

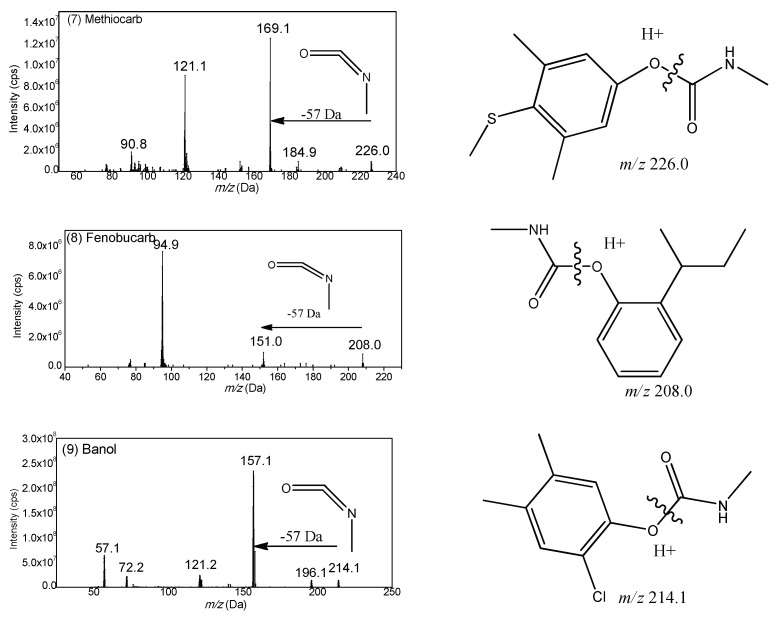
Product ion spectra and proposed fragmentation pathway of nine N-methyl amino formic acid aromatic ester: (**1**) Carbofuran-3-hydroxy; (**2**) Tsumacide; (**3**) Propoxur; (**4**) Carbofuran; (**5**) Carbaryl; (**6**) Isoprocarb; (**7**) Methiocarb; (**8**) Fenobucarb; and (**9**) Banol.

**Figure 5:**
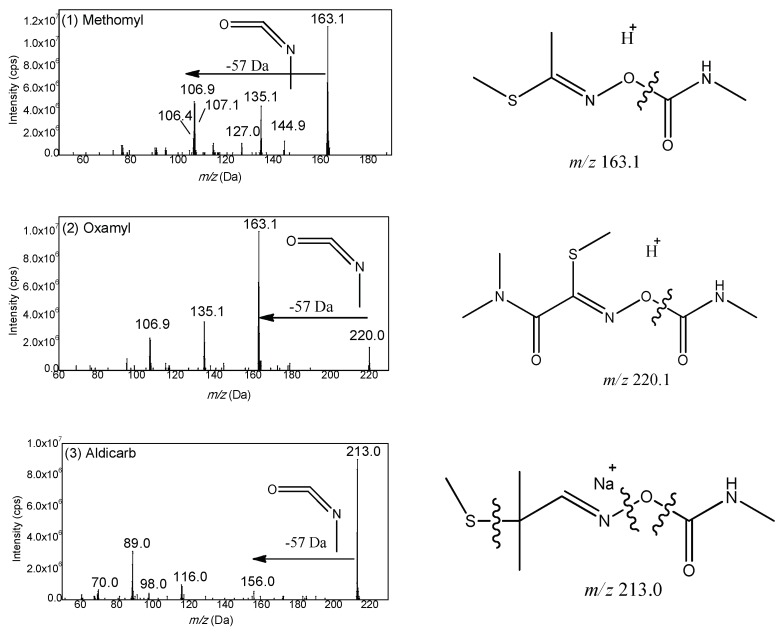

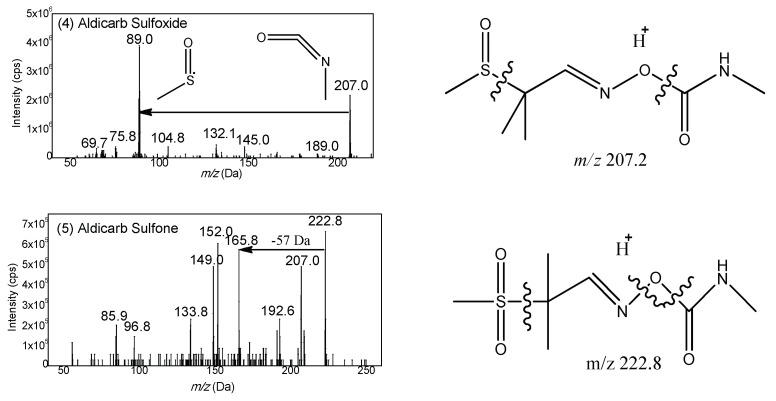
Product ion spectra and proposed fragmentation pathway of three N-methyl amino formic acid oxime ester: (**1**) Methomyl; (**2**) Oxamyl; (**3**) Aldicarb; (**4**) Aldicarb-sulfoxide; and (**5**) Aldicarb-sulfone.

Based on the structure, the fifteen carbamates can be divided into three kinds: N-methyl amino formic acid aromatic ester, N-methyl amino formic acid oxime ester and heterocyclic N-methyl amino formic acid ester. The former two include carbaryl, tsumacide, methiocarb and methomyl; and aldicarb sulfone, aldicarb sulfoxide, etc., respectively, while the latter includes pirimicarb, isolan (highly regulated and unavailable), etc. Generally speaking, in the positive mode of electrospray ionization mass spectrometry, the proton first attaches to the protonation site, and then triggers the cleavages by migrating to the reactive center [36]. With the common structure of amino nitrogen and carbonyl oxygen, the protons could gravitate to these preferred protonation sites, and then trigger the dissociation reaction by migrating to the reactive center. Therefore, the oxygen of ester near the carbonyl carbon is a suitable active site to induce this fragmentation of carbamates. For example, 3-hydroxyl carbofuran belonging to N-methyl amino formic acid aromatic ester, could generate a stable fragment ion *m*/*z* 181.1 by a neutral loss of CH_3_NCO (−57 Da). The resulting spectrum is shown in Figure 3. According to this fragment mechanism, all fifteen carbamate pesticides were investigated and the vast majority of carbamate pesticides, except pirimicarb, could dissolve at the same active site and obtain the same characteristic loss of 57 Da (Figure 4 and Figure 5).

**Figure 6:**
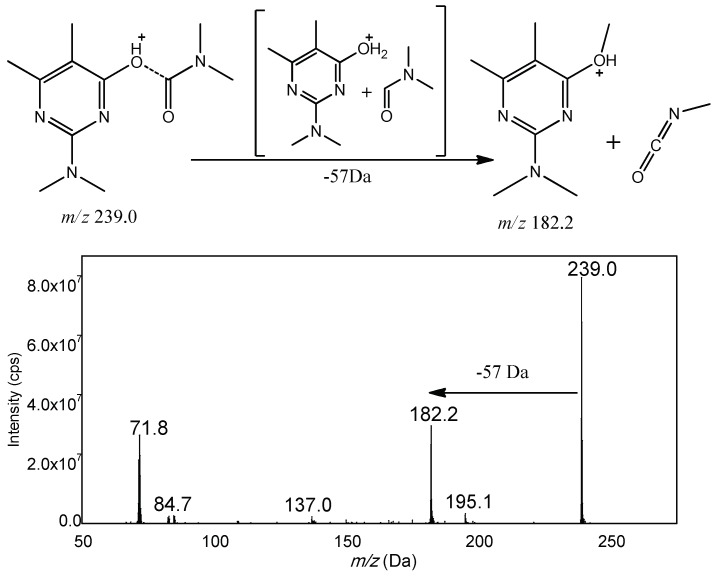
Product ion spectra and probable fragmentation routes of Pirimicarb.

Unexpectedly, 57 Da was lost in pirimicarb. Structurally speaking, it has two methyl groups linked to the amino group and it should produce a loss of 72 Da. In fact, the mass spectrum of pirimicarb indicated an unexpected loss of 57 Da rather than 72 Da, as shown in Figure 6. This is because of the nitrogen-containing pyrimidine heterocyclic ring adjoined the ester oxygen that could induce a methyl transfer reaction. According to a literature survey [37,38,39], the proton is localized at the pyrimidine nitrogen first; and then the charge-remote become the reaction center that forming an ion-neutral complex and induce an alkyl cation transfer.

### 2.4. Method Validation

#### 2.4.1. Matrix Effect

**Figure 7:**
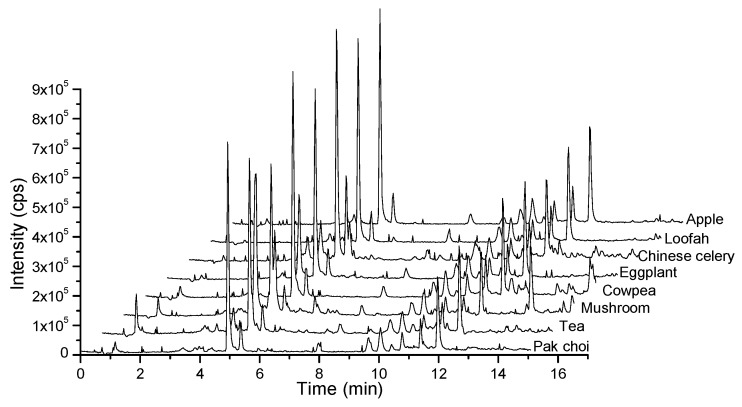
Typical total ion chromatograms of fifteen carbamates at 2 µg/kg spiked in eight matrices (cleaning by 50 mg C18 and 150 mg PSA).

As shown in Figure 7, the complex composition of diverse matrices might have different effects on the accuracy of identification and quantification. It was necessary to correct the matrix effects in LC-MS detection. Eight distinct types of organic vegetable samples or pesticide-free samples were used as matrix-matched blank to evaluate the matrix effects on the ionization of fifteen carbamate residues. The evaluation of matrix effects was calculated using the following equation [40]:

$$\mathrm{ME}=\frac{A_{\mathrm{Matrix}\;}}{A_{S}}\times 100\%$$

where A_Matrix_ is the peak area of the standard solution with the matrix-matched blank and A_S_ is the peak area of the standard solution with initial mobile phase. As shown in Table 3, the percentages of the matrix effects of 15 carbamate pesticides at three different concentrations (2, 20, and 200 ng mL^−1^) ranged 95.4–111.2% (see Table 2 for details). Since there was no obvious ion suppression or ion enhancement at the chosen levels of quantification for 15 carbamate pesticides in eight distinct types of samples, the matrix effect can be ignored. 

#### 2.4.2. Linearity and Analytical Limits

Satisfactory correlation coefficients (R > 0.996) were obtained for fifteen carbamate residues in matrix-matched blank over the concentration range of 0.05–200.0 ng mL^−1^. The analytical limits of the proposed method were measured by the limit of detection (LOD) and the limit of quantification (LOQ) using the following equations [41]:

$$\mathrm{LOD}=C_{S}\frac{3}{S/N}$$

$$\mathrm{LOQ}=C_{S}\frac{10}{S/N}$$

where S/N is the average signal to noise ratio and C_S_ is the concentration of the specific pesticide. The estimated values were tested with a suitable number and kind of samples containing the 15 carbamate pesticides at the corresponding concentrations. All related parameters of the proposed method are summarized in Table 4. The LOD and LOQ were 0.2–2.0 μg kg^−1^ and 0.5–5.0 μg kg^−1^, respectively, which demonstrated a good analytical limit of this method for carbamate residues.

#### 2.4.3. Accuracy and Precision

The accuracy and precision of the method were measured by the recoveries and coefficients of variation (CVs) for intra- and inter-day. Therefore, the standard mixtures solution of fifteen carbamate residues was spiked into distinct types of samples including greengrocery, Chinese celery, loofah, eggplant, cowpea, apple, mushroom and tea leaves, and 24 spiked samples (eight types at three concentrations of 2, 20, and 200 μg kg^−1^) were obtained. All spiked samples were detected three times a day on five different days following the process of Figure 1. The results are shown in the Supplementary Materials. The recovery of fifteen carbamate residues (88.1–118.4%) fell within the recommended Eurachem guidelines of 80–120% [42]. The analysis precision, measured as the coefficient of variation percentage (% CV) of the recovery (3.2–9.8%), was well under the criteria of 10% [42].

### 2.5. Sample Analyses

After validation of the analyti cal methodology through above experimentation, it was used to detect numerous types of samples including apple, carrot, chili pepper, Chinese celery, coriander, cowpea, crown daisy, eggplant, garlic sprout, grape, ginger, leek, lettuce, loofah, mushroom, needle mushroom, orange, pakchoi, radish, shiitake, spinach and tea leaves. Carbofuran and its metabolite Carbofuran-3-hydroxy were the predominant detected residues of the fifteen carbamates. Meanwhile, of the 26 samples (Table S3), eggplant contributed the detected residues of maximum frequency, with concentrations of carbofuran at 85.5, 77.4, 68.4, 35.6, 25.6, 17.3, 16.5 and 11.8 μg kg^−1^, respectively. Pokchoi, spinach, and Chinese celery all had considerable detection rate and concentration of carbofuran. Among all samples, although green tea had the second highest detection rate, the concentration of carbamate residues were localized at relative low concentration of 6.4, 1.5, 1.2, 0.8, 0.6, 0.6, 0.4, 0.3, 0.2 and 0.2 μg kg^−1^, respectively. This was mainly due to the further degradation of carbamate residues in the drying process of tea manufacturing. Cowpea occasionally had the highest concentration of carbofuran at 180.2 μg kg^−1^.

## 3. Materials and Methods

### 3.1. Chemicals and Reagents

Acetonitrile and Methanol were of HPLC grade and obtained from Merck (Darmstadt, Germany). Formic acid (≥98%) was of HPLC grade and purchased from Aladdin (Shanghai, China). High purity water was obtained using a Milli-Q water purification system from Millipore (Bedford, MA, USA). Cleanert^®^ Pesticarb, Cleanert^®^ PSA and Cleanert^®^ C18 for QuEChERS were purchased from Agela Technologies (Beijing, China). Millipore filters of polytetrafluoroethylene (0.22 μm) were purchased from ANPEL Lab (Shanghai, China).

Standards of fifteen carbamates were purchased from Dr. Ehrenstorfer GmbH (Augsburg, Germany): Aldicarb-sulfoxide, Aldicarb-sulfone, Pirimicarb, Carbofuran-3-hydroxy, Methomyl, Oxamyl, Banol, Aldicarb, Metolcarb, Propoxur, Carbofuran, Carbaryl, Isoprocarb, Mercaptodimethur and Fenobucarb. Individual standard solutions were prepared in HPLC-grade methanol at a concentration of 1.0 mg/mL and stored at −28 °C temporarily. 

Samples were collected from local supermarkets, including vegetables (leeks, pakchoi, crowndaisy, coriander, spinach, lettuce, Chinese celery, garlic sprout, loofah, chili pepper, eggplants, cowpea, radish, and turnip), fruits (apples, kiwis, grapes, and oranges), tea (green tea, black tea, and flower tea), and edible fungus (shiitake, mushroom, and needle mushroom). Each fresh sample was crushed and mixed into homogenate, and stored in plastic bottles at −20 °C until analysis.

### 3.2. Instrumentation

HPLC analysis was performed on a Shimadzu LC-30AD system (Shimadzu, Kyoto, Japan) which consist of two interconnected pump units: one with an integrated degasser and the other with a mixer, and is comprised of a UHPLC gradient system, a refrigerated autosampler, and a column oven compartment. Mass spectrometric detection was performed on an AB SCIEX QTRAP^®^ 5500 (AB SCIEX instruments, Foster, CA, USA) in MRM mode and EPI mode. A Turbo V™ Ion Source (ESI) interface in positive ionization mode was used. Both the UHPLC and mass spectrometer were controlled remotely using Analyst^®^ software v. 1.6.2 (AB SCIEX instruments, Foster City, CA, USA). A Waters BEH C18 column (1.7 µm 2.1 mm × 100 mm, Waters, Milford, MA, USA) was applied for analysis. The mass spectrometer was equipped with an electrospray ionization source and spectra were acquired in the positive ion multiple reaction monitoring (MRM) mode and enhanced product ion (EPI) scan modes. MS was optimized using a capillary voltage of 5.50 kV and desolvation temperature of 500 °C. The cone gas pressure and desolvation gas pressure were 50 psi. Nitrogen was used as the cone and collision gasses, respectively. The raw data were analyzed using an Analyst 1.6.2 workstation (AB SCIEX, Foster, CA, USA).

### 3.3. Sample Extraction

Five grams of the homogenized sample was weighed in 50 mL Corning extraction tubes, and 5 mL of acetonitrile was added. The tubes were vibrated vigorously for 20 min in batches by a Multi Reax Vortexer (Heidolph, Schwabach, Germany). After the vortex, 1.0 g of sodium chloride and 2.0 g of sodium sulfate were separately added to each tube for the elimination of moisture, and the tubes were immediately manually shaken vigorously for 1 min to prevent sodium sulfate agglomeration. The process was followed by centrifugation at 8000 rpm for 3 min at 4 °C in a Sigma 2–16 K. centrifuge (Sartorius AG, Göttingen, Germany). The supernatant was collected in 10 mL extraction tubes and subjected to QuEChERS dispersive SPE cleaning. The dSPE agent contained 100 mg of primary secondary amine (PSA), 50 mg of C18EC, and 200 mg of magnesium sulfate. The tubes were vibrated for 30 s and centrifuged in the Sigma 2–16 K centrifuge at 13,000 rpm for 2 min at 20 °C. The supernatants were collected and filtered through 0.22 μm Anpel Syringe Hydrophilic PTFE filters (Anpel, Shanghai, China) before LC-MS/MS analysis.

Organic vegetables were cultivated by Ying Zhou’s mother from her garden. The samples were homogenized with an Ultra-Turrax T 25 homogeniser (IKA^®^ Werke, Staufen, Germany) and extracted using the QuEChERS method.

## 4. Conclusions

In this paper, a quick and credible method to quantify and confirm fifteen carbamate pesticide residues by modified QuEChERS sample pretreatment combined ultra-high performance liquid chromatography–quadrupole-linear ion trap mass spectrometry was systematically developed. For modified QuEChERS part, the composition of the cleaning agents was optimized to adapt the general samples. For the instrumental analysis, based on traditional conformation criteria including selected ion pairs, retention time and relative intensities between transitions from MRM scan mode, the presence of carbamate pesticides in doubtful samples was further confirmed by the matching of carbamate pesticide spectra via EPI scan mode. Then, the fragmentation routes of all the carbamates were preliminarily explained based on the mass spectra that the carbamate could generate a stable characteristic fragment ion by a neutral loss of CH_3_NCO (−57 Da). The linearity, matrix effect, analysis limits, accuracy and repeatability were validated in diverse samples. All fifteen carbamates have good correlation coefficients above 0.996 at the range of 0.05–200 ng mL^−1^. The recoveries of intra- and inter-day experiments were in the range of 80–120% at three concentrations with coefficients of variation all better than 10%. Moreover, the whole process of one dozen samples from pretreatment to final report did not exceed 2 h, which is shorter and exacter than traditional methods. Finally, the method was applied to carbamate residues detection in 26 kinds of samples from local markets, in which carbamates were extensively detected but all below the standard of maximum residue limit. 

## Figures and Tables

**Table 1:** Comparative study between the published analysis methods for carbamates.

Target	Object	Sample Pretreatment	Instrumental Analysis	Analysis Limits	Disadvantage	Ref.
Carbaryl	soil	ionic liquid-dispersive liquid–liquid microextraction	HPLC-FD(fluorescence detector)	0.63–4.0 ng g^−1^	Ionic liquids are not commercially available No confirmation spectra	[11]
Seven carbamates	gram, wheat, lentil, soybean, fenugreek leaves apple	column chromatography	HPLC-UV	0.08–1.16 mg L^−1^	Large amount of Solvents Lack of sensitivity No confirmation spectra	[17]
Seven carbamates	/	/	HPLC post-column derivatization and fluorescence detection	0.2–0.7 ng	No application No confirmation spectra	[26]
Eleven carbamates	/	/	HPLC post-column derivatization and fluorescence detection	0.5 ng mL^−1^	The tempestuously hydrolyzation of standards in dilute hydrochloric acid solution No confirmation spectra	[27]
Fifteen carbamates	corn, cabbage, tomato	QuEChERS method	LC-MS (QDa)	1 µg mL^−1^	Lack of sensitivity; expensive No confirmation spectra	[28]
Thirteen carbamates	orange, grape, onion, tomatoes	Matrix solid-phase dispersion (concentration)	LC-MS (ESI/APCI)	0.001–0.01 mg kg^−1^	No confirmation spectra	[29]
Thirteen carbamates	Traditional Chinese Medicine	QuEChERS method	UPLC-MS (MRM)	5.0–10.0 µg kg^−1^	No confirmation spectra	[30]

**Table 2:** Retention time and MS parameters of the fifteen carbamates pesticides.

No.	Compound	Retention Time (min)	^1^ CAS No.	Precursor Ion (*m*/*z*)	Product Ion (*m*/*z*)	Declustering Potential (V)	Collision Energy (eV)
1	Aldicarb-sulfoxide	3.81	1646-87-3	206.9	132.0 89.0 *	130 130	8 17
2	Aldicarb-sulfone	4.17	1646-88-3	222.8	166.1 * 148.0	140 140	11 18
3	Pirimicarb	5.45	23103-98-2	239.1	182.1 * 72.0	80 80	20 30
4	Carbofuran-3-hydroxy	5.57	16655-82-6	237.8	181.0* 163.0	150 150	15 19
5	Methomyl	5.58	16752-77-5	162.8	135.0 106.0 *	180 180	26 28
6	Oxamyl	5.59	23135-22-0	220.0	72.0 163.1 *	40 40	18 10
7	Aldicarb	6.40	116-06-3	212.7	89.0 * 156.0	150 150	18 19
8	Tsumacide	6.60	1129-41-5	166.0	109.0 *	50	13
9	Propoxur	6.87	114-26-1	210.0	168.0 * 153.0	60 60	18 10
10	Carbofuran	6.98	1563-66-2	221.7	165.0 * 123.0	120 120	15 27
11	Carbaryl	7.04	63-25-2	202.0	145.0 * 117.0	60 80	12 35
12	Isoprocarb	7.18	2631-40-5	194.0	95.0 137.0 *	100 100	19 11
13	Methiocarb	7.33	2032-65-7	226.0	169.0 * 121.0	55 55	12 24
14	Fenobucarb	7.84	3766-81-2	208.0	95.0 * 152.0	70 70	19 11
15	Banol	8.29	671-04-5	214.1	157.1 * 121.2	60 60	12 16

^1^ CAS: chemical abstracts service; *: quantitative ion.

**Table 3:** Matrix effects of the fifteen carbamates pesticides in distinct samples.

Compound	Matrix Effects/% (*n =* 3; 2, 20, 200 ng mL^−^^1^)							
	Pak Choi	Chinese Celery	Loofah	Eggplant	Cowpea	Apple	Mushroom	Tea
Aldicarb-sulfoxide	98.6	98.5	96.3	98.8	96.0	95.4	96.6	97.6
Aldicarb-sulfone	97.8	96.5	98.3	99.1	95.7	96.2	98.4	96.9
Pirimicarb	105.1	102.0	98.0	98.7	96.3	98.9	101.2	99.2
Carbofuran-3-hydroxy	102.3	101.3	103.5	107.5	101.2	102.5	101.0	105.4
Methomyl	110.2	108.6	107.2	108.1	105.4	111.2	105.7	109.2
Oxamyl	102.5	98.6	105.2	106.3	102.0	102.1	105.2	106.2
Aldicarb	107.5	102.1	100.2	101.3	100.8	103.2	100.5	102.3
Tsumacide	105.0	100.9	102.3	98.6	97.9	99.0	102.6	102.7
Propoxur	102.3	105.4	102.7	104.9	101.8	102.4	103.1	101.5
Carbofuran	102.5	105.9	106.2	103.1	98.7	99.2	97.4	98.5
Carbaryl	102.3	104.1	106.5	104.5	101.7	102.4	106.9	107.4
Isoprocarb	102.3	104.6	102.5	104.8	101.2	107.5	108.4	101.8
Methiocarb	105.9	104.1	102.4	106.2	107.1	105.1	106.3	105.1
Fenobucarb	102.1	105.2	102.6	107.8	110.0	108.4	107.6	106.8
Banol	105.6	104.2	102.9	104.1	103.2	102.5	102.3	101.1

**Table 4:** Linear ranges, linear equations, correlation coefficients (R) limits of detection (LODs) and limits of quantification (LOQs) of the fifteen carbamate pesticides.

Compound	Linear Range (ng mL^−1^)	Linear Equation	R (1/X^2^)	LOD (μg kg^−1^)	LOQ (μg kg^−1^)
Aldicarb-sulfoxide	0.05–200	Y = 2.42 × 10^4^X + 812.9	0.9963	0.2	0.5
Aldicarb-sulfone	0.50–200	Y = 1.44 × 10^4^X + 10,476	0.9972	2.0	5.0
Pirimicarb	0.05–200	Y = 5.49 × 10^5^X + 15,110.0	0.9968	0.2	0.5
Carbofuran-3-hydroxy	0.10–200	Y = 1.75 × 10^4^X + 457.2	0.9964	0.3	1.0
Methomyl	0.05–200	Y = 2.91 × 10^4^X + 102,771	0.9962	0.2	0.5
Oxamyl	0.05–200	Y = 8.38 × 10^4^X + 2287.1	0.9965	0.2	0.5
Aldicarb	0.05–200	Y = 1.96 × 10^4^X + 7283.2	0.9973	0.2	0.5
Tsumacide	0.05–200	Y = 6.64 × 10^4^X + 3330.4	0.9967	0.2	0.5
Propoxur	0.05–200	Y = 1.27 × 10^5^X + 3441.9	0.9963	0.2	0.5
Carbofuran	0.20–200	Y = 6.31 × 10^4^X + 543.2	0.9982	0.3	1.0
Carbaryl	0.05–200	Y = 6.01 × 10^4^X + 4445.6	0.9968	0.2	0.5
Isoprocarb	0.05–200	Y = 9.33 × 10^4^X + 15,304	0.9957	0.2	0.5
Methiocarb	0.05–200	Y = 9.86 × 10^4^X + 6565.0	0.9965	0.2	0.5
Fenobucarb	0.05–200	Y = 1.24 × 10^5^X + 7436.7	0.9962	0.2	0.5
Banol	0.05–200	Y = 1.01 × 10^5^X + 2436.7	0.9971	0.2	0.5

Y, peak area; X, mass concentration, ng mL^−1^; 1/X^2^, least square method.

## Supplementary Materials

The following are available online, Figure S1: Recovery tests of different proportions of PSA, GCB, and C18 in multi-matrices, Table S1: Intra-day accuracy and precision (*n =* 3), Table S2: Inter-day accuracy and precision (*n =* 15), Table S3: The maximum residues of 15 carbamate pesticide residues in 26 different samples (*n =* 28).
