# Supplementary material for: Quantification and Confirmation of Fifteen Carbamate Pesticide Residues by Multiple Reaction Monitoring and Enhanced Product Ion Scan Modes via LC-MS/MS QTRAP System
Source: Molecules. 2018 Sep 29;23(10):2496. doi: 10.3390/molecules23102496 (PMC6222809; doi:10.3390/molecules23102496)
Supplement: Supplementary file 1 [file molecules-23-02496-s001.pdf]

Figure S1. Recovery tests of different proportions of PSA and C18 in multiple samples.

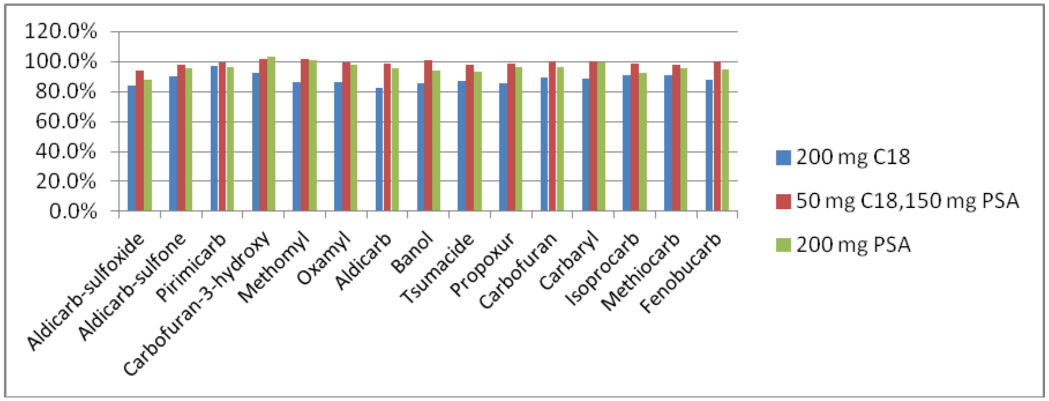

Table S1. Intra-day accuracy and precision (n=3).

Coumpound	Spiked (μg/kg)	Greengrocery		Eggplant		Apple		Mushroom		Tea	
		Recovery (%)	CV (%)	Recover y (%)	CV (%)	Recover y (%)	CV (%)	Recover y (%)	CV (%)	Recover y (%)	CV (%)
Aldicarb-sulfoxide	2	95.6	7.5	102.5	8.8	96.0	5.4	96.6	7.6	92.3	6.3
	20	97.0	4.6	94.0	6.6	96.3	6.9	97.6	6.5	96.0	5.4
	200	103.5	3.7	102.5	4.8	94.1	7.1	96.9	5.6	93.3	7.1
Aldicarb-sulfone	2	97.8	8.2	98.3	9.1	95.7	6.2	98.4	6.9	96.3	6.2
	20	96.8	5.7	99.1	6.5	93.3	5.9	96.7	6.5	95.6	5.7
	200	98.3	6.4	96.3	5.3	92.8	7.3	95.6	4.7	94.4	4.9
Pirimicarb	2	105.1	6.3	98.0	8.7	96.3	8.9	101.2	7.2	98.6	6.5
	20	100.2	7.2	99.6	6.9	98.9	6.5	99.9	5.8	97.3	4.9
	200	109.5	6.0	101.2	5.5	98.8	5.7	97.6	6.3	99.6	4.4
Carbofuran-3-hydroxy	2	102.3	6.3	103.5	6.5	101.2	6.5	101.0	5.4	99.8	5.6
	20	107.5	5.9	107.7	7.1	105.5	8.6	98.8	6.8	97.8	7.3
	200	102.2	8.8	98.5	4.8	98.9	6.2	99.9	4.7	101.1	4.8
Methomyl	2	110.2	5.7	107.2	9.5	105.4	7.2	105.7	7.2	105.2	6.5
	20	108.8	9.0	108.1	6.7	111.2	5.9	102.3	6.5	101.4	7.2
	200	105.4	5.6	100.4	5.5	108.5	4.7	109.2	5.3	98.8	4.8
Oxamyl	2	102.5	5.3	103.2	6.3	101.0	5.1	105.2	6.2	99.7	6.0
	20	105.2	6.1	102.0	5.2	99.7	6.3	100.7	5.4	98.6	5.9
	200	106.3	7.0	102.1	4.8	106.0	4.8	98.8	6.1	95.6	4.7
Aldicarb	2	107.5	5.9	98.8	6.3	100.8	3.2	100.5	4.3	99.0	4.5
	20	100.2	6.1	97.4	5.9	99.0	5.6	100.2	5.1	98.7	6.2
	200	101.3	4.8	96.5	4.4	97.4	4.7	103.3	3.9	102.1	6.3

Tsumacide	2	105.0	6.0	102.3	5.6	97.9	6.0	102.6	3.7	105.5	6.6
	20	99.7	4.7	98.8	4.3	98.8	5.6	96.7	5.7	109.8	4.2
	200	98.4	5.0	97.6	5.0	98.0	4.7	97.8	4.5	103.3	3.8
Propoxur	2	102.3	6.3	102.7	4.9	101.8	4.4	103.1	7.5	101.3	6.3
	20	98.7	5.2	100.2	5.3	105.8	5.6	105.8	6.0	92.2	5.7
	200	96.8	4.9	97.3	7.6	104.4	3.9	106.6	7.2	94.8	4.9
Carbofuran	2	102.5	6.0	106.2	3.1	98.7	9.2	97.4	8.5	102.7	5.9
	20	89.5	8.7	99.6	3.3	99.8	8.5	98.7	9.6	105.6	4.1
	200	96.8	5.6	98.7	4.8	97.7	6.5	95.6	9.4	103.5	6.3
Carbaryl	2	102.3	6.6	106.5	5.6	101.7	5.4	106.9	7.4	104.5	6.0
	20	104.3	5.8	105.6	6.8	102.4	6.3	99.8	6.3	96.5	4.9
	200	101.1	4.7	102.2	5.8	108.1	7.0	93.3	9.0	95.8	5.3
Isoprocarb	2	102.3	6.1	102.5	5.0	101.2	7.1	108.4	4.8	99.8	6.0
	20	102.5	5.2	103.2	4.7	107.5	4.9	105.8	6.9	98.7	4.9
	200	100.7	4.3	99.8	5.3	106.8	5.1	106.2	7.3	96.7	6.3
Methiocarb	2	99.5	3.9	102.4	4.9	107.1	4.2	106.3	5.1	106.2	4.5
	20	105.6	8.0	96.8	3.8	105.1	3.9	105.5	4.8	101.8	6.3
	200	98.8	5.5	97.1	5.7	108.6	8.4	104.7	6.7	110.7	6.0
Fenobucarb	2	99.1	7.1	102.6	6.8	110.0	6.3	107.6	6.8	107.8	8.3
	20	98.6	6.8	98.8	6.6	107.7	6.0	106.6	5.6	105.6	5.7
	200	97.3	5.9	93.5	5.8	108.4	5.9	108.3	4.8	109.8	4.6
Banol	2	105.6	5.3	102.9	4.1	103.2	5.2	102.3	5.1	101.7	5.1
	20	104.1	4.2	103.3	5.2	101.1	4.7	100.2	4.6	104.5	4.9
	200	102.5	7.0	98.7	6.8	97.8	6.0	99.7	3.8	103.4	8.0

Table S2. Inter-day accuracy and precision (n=15).

Coumpound	Spiked (μg/kg)	Greengrocery		Eggplant		Apple		Mushroom		Tea	
		Recovery (%)	CV (%)	Recover y (%)	CV (%)	Recover y (%)	CV (%)	Recover y (%)	CV (%)	Recover y (%)	CV (%)
Aldicarb-sulfoxide	2	93.7	9.7	94.8	9.8	96.0	9.5	96.6	9.6	96.5	9.8
	20	94.0	8.4	95.7	7.5	94.5	8.6	93.7	8.8	93.2	8.5
	200	102.5	6.3	102.5	4.7	96.4	7.4	92.4	8.4	91.8	9.4
Aldicarb-sulfone	2	97.8	8.5	93.3	9.1	93.5	9.6	98.4	9.6	98.0	9.0
	20	100.0	7.4	96.4	7.7	95.7	8.0	90.1	8.7	94.7	5.6
	200	110.7	6.6	101.0	6.9	98.1	7.3	92.3	6.6	90.5	4.3
Pirimicarb	2	99.2	9.0	98.0	9.3	96.3	9.1	101.2	9.2	93.6	8.7
	20	106.9	8.1	98.7	8.6	99.4	7.2	89.6	8.7	96.5	6.8
	200	111.1	6.7	101.5	7.5	100.7	4.1	92.6	6.3	98.8	5.2
Carbofuran-3-hydroxy	2	101.9	7.6	99.9	9.5	101.2	8.6	101.0	9.5	105.4	9.0
	20	102.2	6.8	101.8	7.4	104.0	7.1	98.7	8.2	110.2	8.8
	200	118.4	6.6	103.4	6.8	96.6	6.4	95.6	5.8	95.8	6.6
Methomyl	2	101.3	8.8	97.2	9.1	105.4	8.1	105.7	8.9	109.2	8.6
	20	96.2	7.3	98.7	6.8	95.9	7.9	106.2	6.5	90.5	7.9
	200	115.6	5.9	105.1	6.6	98.8	5.4	89.9	6.2	96.2	6.3
Oxamyl	2	99.0	8.5	98.1	9.3	102.0	9.1	105.2	8.9	98.8	8.9
	20	96.2	7.9	99.2	8.9	101.4	7.3	99.7	7.5	95.6	7.8
	200	112.7	6.4	110.4	5.6	105.0	6.3	96.5	6.3	96.0	6.4
Aldicarb	2	98.9	9.3	100.2	9.7	100.8	9.0	95.6	9.8	98.9	6.8
	20	92.3	8.5	102.5	8.5	98.9	8.8	89.7	8.9	89.9	8.3
	200	110.6	5.2	104.8	6.9	96.7	5.4	88.1	8.3	90.6	7.6

Tsumacide	2	98.4	8.7	98.6	9.9	97.9	8.8	102.6	8.8	90.6	9.6
	20	95.1	6.9	105.7	7.8	105.4	7.1	96.5	7.4	89.9	7.8
	200	111.2	5.3	105.2	6.4	104.1	3.6	97.1	6.4	90.0	5.5
Propoxur	2	99.7	9.5	102.7	9.5	101.8	8.9	103.1	8.8	93.4	6.5
	20	99.2	8.4	104.9	8.9	99.7	5.4	98.2	8.7	98.1	9.0
	200	111.1	5.8	102.5	7.7	102.2	6.7	95.5	6.3	97.0	4.7
Carbofuran	2	99.8	9.4	103.1	9.6	98.7	9.6	97.4	9.7	97.3	9.6
	20	98.6	8.1	98.6	8.0	101.6	6.0	95.4	8.3	98.8	8.5
	200	114.0	6.0	106.2	7.5	98.7	7.1	90.0	6.6	89.7	6.1
Carbaryl	2	98.9	9.4	97.4	9.8	101.7	8.7	106.9	9.4	97.7	9.8
	20	100.6	8.3	101.7	8.6	98.2	6.8	105.7	7.7	90.2	8.5
	200	107.6	6.7	104.5	6.5	97.4	4.9	106.8	6.1	88.9	7.7
Isoprocarb	2	98.1	9.3	99.0	9.8	101.2	8.9	98.4	8.6	88.7	9.2
	20	101.1	8.2	100.2	8.3	100.1	7.4	108.2	9.0	96.0	9.6
	200	110.7	6.5	105.6	6.6	97.6	6.2	95.4	5.8	89.7	8.7
Methiocarb	2	95.6	9.6	96.4	9.2	107.1	9.7	106.3	9.0	98.1	9.4
	20	98.1	8.4	99.2	7.8	102.7	7.6	95.6	9.1	89.6	8.6
	200	101.8	5.9	100.5	6.4	97.1	4.8	90.7	5.0	97.5	6.5
Fenobucarb	2	99.9	8.9	97.6	9.7	110.0	9.3	107.6	8.7	90.2	9.7
	20	97.7	7.2	98.5	8.8	101.8	7.8	106.8	6.9	92.2	8.4
	200	109.5	8.0	100.7	6.4	94.4	6.5	98.5	5.3	94.0	6.8
Banol	2	97.6	9.8	98.5	9.4	103.2	9.6	102.3	9.3	93.3	9.5
	20	97.2	7.5	98.9	5.8	101.5	7.7	98.4	8.5	98.4	8.7
	200	108.1	6.1	104.5	5.4	99.7	5.1	93.3	7.0	96.1	6.4

Table S3. The maximum residues of 15 carbamate pesticide residues in 26 different samples (n=28).

Compound	Freq.[1] (of 28)	Residual Concentration (µg kg-1)														
		Aldicarb sulfoxide	Aldicarb sulfone	Pirimicarb	Carbofuran 3-hydroxy	Methomyl	Oxamyl	Aldicarb	Tsumacide	Propoxur	Carbofuran	Carbaryl	Isoprocarb	Methiocarb	Fenobucarb	Banol
Apple	1	ND	ND	ND	ND	ND	ND	ND	ND	ND	1.6	ND	ND	ND	ND	ND
Carrot	1	ND	ND	ND	ND	ND	ND	ND	ND	ND	3.2	ND	ND	ND	ND	ND
Cabbage	5	ND	ND	ND	23.0	ND	ND	ND	ND	1.3	59.3	15.6	ND	ND	ND	ND
Chilli pepper	3	ND	ND	ND	3.6	ND	ND	ND	ND	ND	32.1	ND	ND	ND	1.6	ND
Chinese celery	6	ND	ND	ND	ND	ND	ND	ND	ND	ND	36.5	ND	ND	ND	0.8	ND
Chinese yam	2	ND	ND	ND	1.5	ND	ND	ND	ND	ND	6.7	ND	ND	ND	ND	ND
Coriander	2	ND	ND	ND	3.6	ND	ND	ND	ND	ND	74.6	ND	ND	ND	ND	ND
Cowpea	4	ND	ND	ND	39.9	ND	ND	ND	ND	ND	180.2	ND	ND	ND	ND	ND
Crown daisy	4	ND	ND	37.7	13.6	ND	ND	ND	ND	0.3	25.6	ND	ND	ND	ND	ND
Eggplant	15	42.2	ND	ND	28.1	ND	ND	ND	ND	ND	85.5	ND	ND	ND	ND	ND
Garlic sprout	1	ND	ND	ND	ND	ND	ND	ND	ND	ND	1.8	ND	ND	ND	ND	ND
Grape	1	ND	ND	25.6	ND	ND	ND	ND	ND	ND	ND	ND	ND	ND	ND	ND
Ginger	4	ND	ND	ND	18.8	ND	ND	ND	ND	ND	45.8	ND	ND	ND	ND	ND
Leek	1	ND	ND	ND	ND	ND	ND	ND	ND	ND	2.6	ND	ND	ND	ND	ND
Lettuce	4	ND	ND	36.6	23.3	ND	ND	ND	ND	ND	23.0	ND	ND	ND	ND	ND
Loofah	4	ND	ND	ND	ND	ND	ND	ND	ND	ND	ND	ND	ND	ND	ND	ND
Mushroom	1	ND	ND	ND	1.5	ND	ND	ND	ND	ND	ND	ND	ND	ND	ND	ND
Needlemushr	1	ND	ND	ND	3.7	ND	ND	ND	ND	ND	ND	ND	ND	ND	ND	ND
Orange	3	ND	ND	3.4	3.5	ND	ND	ND	ND	ND	13.8	ND	ND	ND	ND	ND
Pak choi	8	ND	ND	9.7	23.7	ND	ND	ND	36.6	ND	69.8	ND	ND	ND	ND	ND
Pear	3	ND	ND	ND	10.7	ND	ND	ND	ND	ND	12.2	ND	ND	ND	ND	ND
Radish	0	ND	ND	ND	ND	ND	ND	ND	ND	ND	ND	ND	ND	ND	ND	ND
Shii-take	0	ND	ND	ND	ND	ND	ND	ND	ND	ND	ND	ND	ND	ND	ND	ND

Spinach	8	ND	ND	7.7	19.8	50.7	ND	ND	ND	ND	88.4	ND	ND	ND	ND	ND
Tea (Green)	10	ND	ND	0.4	1.2	ND	ND	ND	ND	ND	6.4	1.5	ND	ND	ND	ND
Tomato	1	ND	ND	6.7	ND	ND	ND	ND	ND	ND	ND	ND	ND	ND	ND	ND

Freq.[1]: the frequency of detected concentration higher than the LOQs of total 26 samples/type
